# Inhibition of gap junctional Intercellular communication in WB-F344 rat liver epithelial cells by triphenyltin chloride through MAPK and PI3-kinase pathways

**DOI:** 10.1186/1745-6673-5-17

**Published:** 2010-06-30

**Authors:** Chung-Hsun Lee, I-Hui Chen, Chia-Rong Lee, Chih-Hsien Chi, Ming-Che Tsai, Jin-Lian Tsai, Hsiu-Fen Lin

**Affiliations:** 1 Department of Emergency Medicine, National Cheng Kung University Hospital, Tainan, Taiwan; 2 Graduate Institute of Occupational Safety and Health, College of Health Science, Kaohsiung Medical University, Kaohsiung 80708, Taiwan; 3 Department of Ophthalmology, Chang Gung Memorial Hospital, Kaohsiung, Taiwan; 4 Chang Gung University, College of Medicine, Kaohsiung, Taiwan

## Abstract

**Background:**

Organotin compounds (OTCs) have been widely used as stabilizers in the production of plastic, agricultural pesticides, antifoulant plaints and wood preservation. The toxicity of triphenyltin (TPT) compounds was known for their embryotoxic, neurotoxic, genotoxic and immunotoxic effects in mammals. The carcinogenicity of TPT was not well understood and few studies had discussed the effects of OTCs on gap junctional intercellular communication (GJIC) of cells.

**Method:**

In the present study, the effects of triphenyltin chloride (TPTC) on GJIC in WB-F344 rat liver epithelial cells were evaluated, using the scrape-loading dye transfer technique.

**Results:**

TPTC inhibited GJIC after a 30-min exposure in a concentration- and time-dependent manner. Pre-incubation of cells with the protein kinase C (PKC) inhibitor did not modify the response, but the specific MEK 1 inhibitor PD98059 and PI3K inhibitor LY294002 decreased substantially the inhibition of GJIC by TPTC. After WB-F344 cells were exposed to TPTC, phosphorylation of Cx43 increased as seen in Western blot analysis.

**Conclusions:**

These results show that TPTC inhibits GJIC in WB-F344 rat liver epithelial cells by altering the Cx43 protein expression through both MAPK and PI3-kinase pathways.

## Background

Organotin compounds have been widely used as agricultural biocides, antifouling agents in boat paint, wood preservatives, and stabilizers for polyvinylchloride polymers (PVC) in industry [1,2]. Triphenyltin (TPT) is an organotin compound which is widely used as fungicides on major food and food-stock crops. It is also used in anti-fouling paints to prevent growth of barnacles and other fouling organisms on boats and ships [3]. Organotin compounds are known to be endocrine disruptors in marine species and may be mahuman beings [4,5]. Tissue concentrations of TPT were correlated with the degree of imposex in rock shells [6,7]. TPT compounds have embryotoxic, myotoxic, genotoxic and immunotoxic effects in mammals [8-11]. The organotin compounds might be incorporated in the most abundant phospholipid of eukaryotic membrane and caused toxicity [12]. Some toxic effects have been observed in aquatic and terrestrial organisms exposed to TPT, such as increased tumor incidence and immune suppression [13,14]. Some studies have revealed that TPT might inhibit the cytotoxic function of human natural killer cells and triphenyltin hydroxide produced tumors in rats and mice [14-16].

Connexins (Cxs) are a group of at least 20 highly conserved proteins that provide the basis for communication through the direct exchange of ions, nutrients, second messengers, electrical coupling, and small metabolites from one cell to its neighboring cells [17-20]. Cell proliferation, differentiation, apoptosis and adaptive responses of differentiated cells can occur as a consequence of the up- or down-regulation of GJIC [21-23]. Disruption in GJIC may cause loss of homeostatic and cell growth control [18,24-26]. Growing evidence suggests that connexin 43 (Cx43), a major gap junction protein, functions as a tumor suppressor gene. Expression of Cx43 is often decreased in human tumor cells and tissues, including those involved in human mammary carcinoma, prostate cancer, human glioblastoma, skin squamous cell carcinoma, lung cancer, esophagus cancer, adrenocortical tumors, ovarian carcinoma, cervical cancer, endometrial carcinoma, and human mesothelioma [27-37]. It has been assumed that using pharmacological stimulation to efficiently restore GJIC in tumor cells might represent a strategy for anti-neoplastic therapies [38-42].

The carcinogenicity of TPT remained unclear. The present work was undertaken to define the effects of TPTC on GJIC in WB-F344 rat liver epithelial cells.

## Materials and methods

### Chemicals

Powder of TPTC was supplied by MERCK (Darmstadt, Germany).

Lucifer yellow, DMSO (dimethylsulfoxide), formaldehyde, MTT (3-[4,5-dimethyl-2-thiazolyl]-2,5-diphenyl-2H-tetrazolium bromide) were supplied by Sigma-Aldrich (St. Louis, MO, USA). D medium and newborn calf serum were from Gibco (Invitrogen cooperation, CA, USA), Trizole was from Invitrogen Life Technologies (Rockville, MD, USA) and 2 X SYBR green PCR master mix was from Applied Biosystems (Foster, CA, USA). The protein kinase C (PKC) inhibitor GF109203X, extracellular signal-regulated protein kinase (ERK) inhibitor PD98059 and PI3 kinase inhibitor LY294002 were from Sigma (St. Louis, MO, USA). Immobilon Western HRP Substrate Peroxide Solution and luminal reagent were supplied by Millipore Corporation (Billerica, MA). All chemicals used in the study were of the highest available purity.

### Cell culture and treatment with chemicals

WB-F344 rat liver epithelial cells [43] were cultured in D medium supplemented with 5% fetal bovine serum and 1% [v/v] penicillin/streptomycin antibiotic. The cells were grown at 37°C in a 5% CO_2 _incubator before being used in the different experiments. Confluent cells, grown in plates, were exposed to various concentrations of TPTC. To prepare the TPTC stock solution, 0.01 g of TPTC powder was dissolved in 10 ml DMSO and then diluted to a final concentration of 1000 ppm.

### Cell toxicity assay of TPTC

The effect of TPTC on the survival of WB F344 cells was assessed using MTT toxicity assay as described previously [44]. In brief, the cells were plated in 100 μl media in 48-well plates (1 × 10^4^/well). On the following day, the experimental medium containing different TPTC concentrations (0, 0.25, 0.5, 1, 2, 3, 4, and 5 ppm) was added, and then incubated for 30 and 60 minutes. Fifty μl of MTT solution (2 mg/ml in PBS) was added to each well and incubated for 6-8 hours. After careful removal of the medium, 150 μl of DMSO was added to each well, and then after careful shaking, the absorbance was read at 570 nm using an ELISA microplate reader (Zenyth 200rt with ADAP software, Anthos Labtec Instruments, Autria). Cell viability was expressed as a percentage of control cells not treated with TPTC and was designated as 100%.

### Colony forming-efficiency assay

Colony forming-efficiency experiments were performed as previously described [45]. In brief, exponentially growing cells were plated at 500 cells/100 mm tissue culture dish in 10 ml D medium, treated with different concentrations of TPTC. Following treatment, the plates were washed two times with the medium. The medium was not replaced, and colonies were fixed and stained after 14 days in culture by water: addition of methanol (1:1) containing crystal violet (1 g/l). Colonies with cell clusters containing more than 50 cells were counted under a dissecting microscope. Data indicate survival as a percentage relative to untreated cells.

### GJIC inhibition assay

GJIC assay was carried out in 35 × 10 mm tissue culture dishes with 100% confluent monolayer cells grown in 2 ml D-medium supplemented with 5% newborn calf serum, 100 U/ml penicillin and streptomycin 100 μg/ml. GJIC was detected using the scrape-loading and dye transfer (SL/DT) technique developed by el-Flouly [46]. Assays for different treatments and vehicle control were run in triplicate in cell culture dishes. Monolayer cells with 100% confluence were incubated with target compounds. For dose-dependent inhibition of GJIC, we treated cells with 0.5, 1.0, 1.5 and, 2.0 ppm TPTC for 30 min. For time-dependent inhibition of GJIC, analysis was performed with 1.5 ppm TPTC for 15, 30, 45, and 60 min. After exposure to the target compounds, the cells were rinsed three times with PBS and 1 ml of lucifer yellow solution was then added to the cell cultures and scrape-loaded with several scrapes using a steel surgical blade. The dye solution was left on the cell cultures for 3 min, and then discarded. The cell cultures were carefully rinsed three times with PBS to remove detached cells and background fluorescence. Several drops of 4% formalin in PBS were added to fix the cell cultures. An inverted fluorescence microscope equipped with a digital camera (Nikon Eclipse TE 2000-U system, Nikon ACT-1 version 2.62, Nikon Corporation, Japan) was employed to record the migration of the lucifer yellow dye from the edge cells of the scrape. The migration was measured on the micrograph. An average value of 30 measurements for each treatment (10 measurements per dish) was regarded as the migration of dye in the cell cultures. The percentage of migration of dye in cell cultures exposed to target compounds to the migration of dye traveling in the vehicle control was employed to evaluate the inhibition of GJIC. For inhibition studies, cultures were pre-incubated for 30 min with various pathway inhibitors prior to treatment with 1.5 ppm TPTC for 30 min.

### Western blot analysis

WB F344 liver cells were treated with TPTC of 1.5 ppm for 15 and 30 min. After treatment, the medium was removed and cells were washed twice with PBS and lysed with 0.5% SDS. Lysates were stored at -80°C. Cell lysates were sonicated, and protein levels were determined using a protein detection assay (BioRad). Sample blue buffer (30% sucrose, 10% SDS, 0.1% bromophenol blue, and 0.2% dithiothreitol) was added and the samples were heated for 10 min at 100°C and loaded onto gels (10% SDS-PAGE). SDS-PAGE-separated proteins were blotted onto a PVDF membrane (Immobilon-PSQ, Millipore, Bedford, MA) using a semi-dry blotter (VWR), and the membrane was blocked with 5% milk in PBS-T buffer [1000 ml PBS with 1 ml Tween 20 (pH 7.4)] for more than 1 h at room temperature. The protein was probed with antibodies (Mouse IgG, Zymed) against connexin 43 at 4°C overnight and this was followed by incubation with horseradish peroxidase-conjugated secondary antibodies (Mouse anti-Goat IgG-HRP, Sigma). Protein visualization was carried out using an enhanced chemiluminescence kit (Pierce) according to the manufacturer's protocol.

### Immunofluorescence staining

Immunofluorescence staining experiment s were performed as previously described[47]. In brief, WB F344 liver cells were plated in 100 μl media in 12 well-plates treated with 1.5 ppm TPTC for 30 min. After treatment, the medium was removed and sections were washed with PBS. 4% paraformaldehyde was added and washed sections with PBS 20 min later. 0.5% triton X-100 (Sigma) was added for 20 min and washed out with PBS. After treatment, diluted primary antibodies mouse IgG against connexin 43 (Santa Cruz Biotechnology, Inc.) with 4% triton X-100 was added and incubated sections for 1 h at room temperature. The sections were washed with PBS, and diluted mouse IgG secondary antibody (Alexa Fluor555 &488) with 4% triton X-100 was added and incubated sections for 1 h at room temperature. After treatment, 4,6'-diamidino-2-phenylindole (DAPI)(Sigma) was added and incubated sections for 10 min at room temperature. An inverted fluorescence microscope equipped with a digital camera (Nikon Eclipse TE 2000-U system, Nikon ACT-1 version 2.62, Nikon Corporation, Japan) was employed to record the fluorescent intensity of the cells.

### Statistical analysis

Means ± SEM were calculated and the data are presented as a percentage of control. All data were analyzed by Sigma Plot 8.0 software using repeated measures. ANOVA (SPSS for window version 12.0.1; SPSS, Inc., Chicago, IL) was performed to examine the effect of independent variables (treatment, day, incubation time, time point). Tests for contrasts were carried out to compare the different levels of the independent variables. P values ≤ 0.05 were considered statistically significant.

## Results

TPTC dissolved easily in DMSO but not in water. To exclude the toxic effects of DMSO on cell viability and diffusion length of GJIC, tests involving exposure to DMSO were carried out. Results revealed that after exposure to 2% DMSO for 30 minutes, the diffusion length of GJIC did not obviously decrease as compared with that of the control group (p > 0.05).

### Cytotoxicity of TPTC

**Figure 1:**
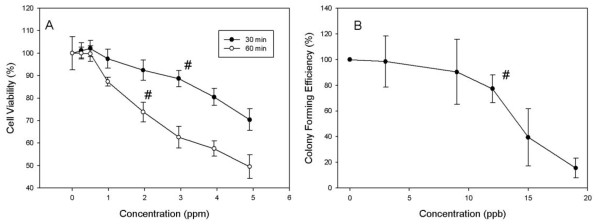
**Cytotoxicity of TPTC in WB-F344 liver cell**. (A) Cell viability of WB-F344 liver cells after exposure to TPTC of different concentrations for 30 min and 60 min. LC50 of TPTC in WB-F344 liver cells amounted to 5 ± 0.9 ppm, n = 5. (B) Colony-forming efficiency of WB-F344 cells treated with different concentrations of TPTC. When the concentration of TPTC was 12 ppb, the proliferation of WB-F344 liver cells was significantly inhibited. Data indicate survival as a percentage relative to untreated cells. All values are represented as means ± S.D. of five independent experiments. Statistical significance was determined using ANOVA (*p < 0.05).

Cytotoxicity evoked by TPTC in WB-F 344 cells was tested with 0, 0.25, 0.5, 1, 2, 3, 4, and 5 ppm of TPTC using the MTT proliferation assay. After 30- and 60-min exposure to TPTC, it was found that cell viability decreased obviously with increasing concentration of TPTC and the lethal concentration 50 (LC 50) in 60 min calculated was 5 ppm (Fig. 1A.)

Colony-forming efficiency in WB-F 344 cells was evaluated using TPTC of 0, 3, 9, 12, 15, 18 ppb. After 14 days of exposure, the colony-forming efficiency decreased significantly when TPTC concentration exceeded 12 ppb (Fig. 1B.)

### Dose- and time- dependent inhibition of GJIC by TPTC

**Figure 2:**
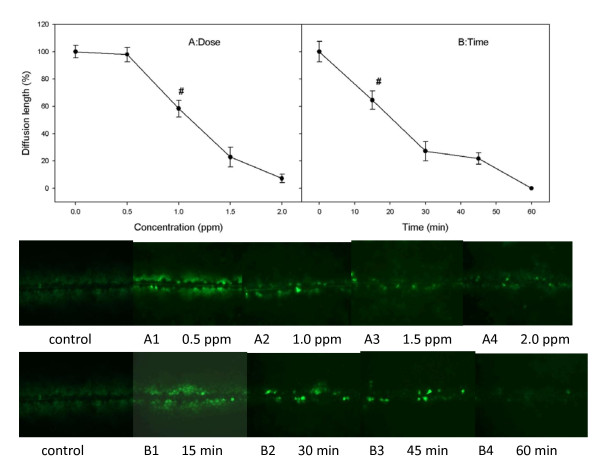
**Inhibition of GJIC by TPTC using the modified scrape-loading/dye transfer method with the Lucifer yellow fluorescent dye**. (A) Dose-dependent inhibition of GJIC after 30-min TPTC exposure. (B) Time-dependent inhibition of GJIC exposed to TPTC. Cells were treated with 1.5 ppm TPTC. The results are represented as means ± S.D. of at least three independent experiments. Statistical significance was determined using ANOVA (*p < 0.05).

Inhibition of GJIC has been suggested to be an important activity of tumor promoters [36]. Therefore, the capacity of TPTC to inhibit GJIC was measured in concentrations with 0.5, 1.0, 1.5 and 2 ppm TPTC after 30 min of exposure. As shown in Figure 2A, TPTC inhibited significantly GJIC in WB-F344 liver cells. The migration of Lucifer yellow dye in scraped WB F344 liver cells treated with TPTC was less than that in untreated cells, when the concentration was 1.0 ppm (*p < 0.05).

The effects of TPTC on GJIC were evaluated with cells exposed to TPTC for 15 min, 30 min, 45 min, and 60 min. After 15 min of exposure to 1.5 ppm of TPTC, the diffusion length was significantly decreased as compared with that of the control group (p < 0.05) (Fig. 2B). The diffusion length reduced gradually with time and became almost invisible after 60 min of exposure to 1.5 ppm of TPTC.

### Effects of PKC, ERK and PI3 kinase on GJIC response

**Figure 3:**
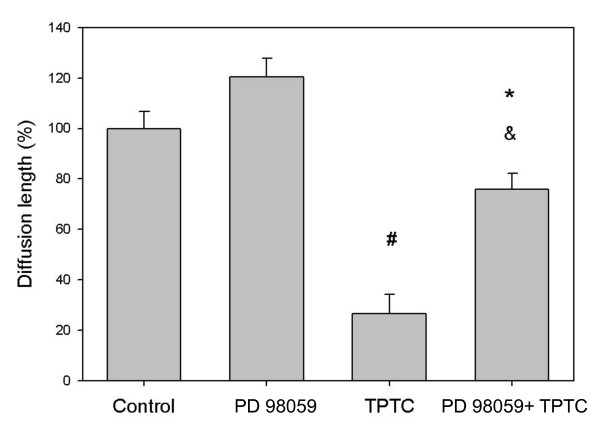
**Effect of PD98059 (MEK 1 inhibitor) on TPTC-induced disruption of GJIC in WB-F344 cells (mean values ± S.D.)**. The control group comprises negative controls. The PD98059 group comprises cells treated with 50 μM PD98059 for 30 min. The TPTC group comprises cells treated with 1.5 ppm TPTC for 30 min. The PD98059 + TPTC group comprises cells pre-treated with 50 μM PD98059 for 30 min and then exposed to 1.5 ppm TPTC for 30 min. Asterisks indicate statistically significant difference. (^# ^p < 0.05 compared with the control group, *p < 0.05 compared with the group exposed to PD98059 alone, and ^&^p < 0.05 compared with the group exposed to TPTC alone.)

Organotin compounds showed that inhibition through some kinase pathways is a possible mechanism involved in the apoptotic effects [48]. The mitogen-activated protein kinase (MAPK) pathway has been shown to be involved in the inhibition of GJIC by TPA [49-54]. Its role in the TPTC-induced inhibition of GJIC was studied next. No specific inhibitor of MAPK was available, but PD98059, a MEK1 inhibitor that blocks ERK activation, was used as an inhibitor of the pathway [55-57]. MEK 1 is the direct upstream activator kinase of MAPKs. The cells were pre-exposed to 50 μM PD98059 for 30 min prior to co-exposure to TPTC (1.5 ppm) for 30 min The scrape-loading assays were then repeated using the ERK inhibitor PD98059. The data showed that PD98059 restored significantly GJIC in TPTC-treated liver cells (p < 0.05) (Fig. 3), Thus, the MAPK signaling pathway was clearly involved in the inhibition of GJIC by TPTC.

**Figure 4:**
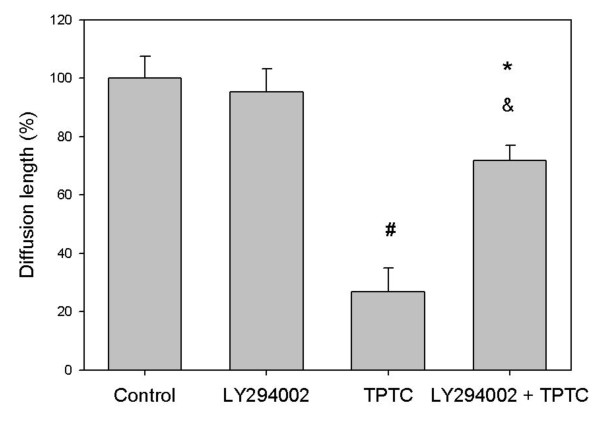
**Effect of LY294002 (PI3K inhibitor) on TPTC-induced disruption of GJIC in WB-F344 cells (mean values ± S.D.)**. The control group comprises negative controls. The LY294002 group comprises cells treated with.100 μM LY294002 for 30 min. The TPTC group comprises cells treated with 1.5 ppm TPTC for 30 min. The LY294002 + TPTC group comprises cells pre-treated with 100 μM LY294002 and exposed to 1.5 ppm TPTC for 30 min. Asterisks indicate statistically significant difference. (# p < 0.05 compared with the control group, * p < 0.05 compared with the group exposed to LY294002 alone, and p < 0.05 compared with the group exposed to TPTC alone.)

Phosphatidylinositol 3'-kinase (PI3K) has been demonstrated to be critical in mediating several aspects of PDGF actions in various cells [23,58-62]. To explore the potential role of PI3K signaling in the signaling processes involved in TPTC-induced disruption of GJIC in liver cells, we measured GJIC in rat liver cells with and without pre-treatment with the Pl3K inhibitor LY294002 (100 μmol/L) before exposure to TPTC (1.5 ppm) for 30 min. As shown in Fig. 4, pre-incubation of rat liver cells with LY294002 (100 μmol/L) for 30 min almost stopped completely the inhibition of GJIC caused by TPTC, although the inhibitor itself did not exert much influence on GJIC, as compared with the control. Similar result was also found in the group exposed to TPTC and PD98059 as compared with that exposed to TPTC alone (Fig. 3). Thus, we conclude that TPTC blocked GJIC through MAPK and PI3K pathways.

**Figure 5:**
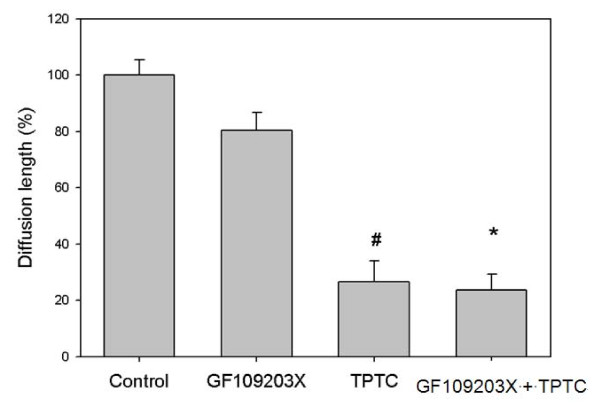
**Effect of GF109302X (PKC inhibitor) on TPTC-induced disruption of GJIC in WB-F344 cells (mean values ± S.D.)**. The control group comprises negative controls. The GF109302X group comprises cells treated with 10 μM GF109203X for 30 min. The TPTC group comprises cells treated with 1.5 ppm TPTC for 30 min. The GF109302X + TPTC group comprises cells pre-treated with 10 μM GF109203X for 30 min and then exposed to 1.5 ppm TPTC for 30 min. Asterisks indicate statistically significant difference. (# p < 0.05 compared with the control group, *p < 0.05 compared with the group exposed to GF109302X alone.)

To study the involvement of protein kinase C (PKC) in the inhibition of GJIC by TPTC, an inhibitor of PKC, GF109203X (bisindolylmaleimide 1) was utilized to block the activity of the enzyme before exposure to TPTC-GF109203X inhibits the isozymes of PKC α, β_I_, β_II_, γ, δ, and ε [63,64]. The cells were pre-exposed to the PKC inhibitor (10 μM) for 30 min prior to co-exposure to TPTC (1.5 ppm) and incubated further for 30 min. The diffusion length of GJIC did not obviously decrease when only GF109203X was added. On the other hand, cells were treated with 10 μM GF109203X for 30 min, followed by addition of TPTC. The diffusion length of GJIC decreased obviously following the addition of TPTC or TPTC with GF109203X (Fig. 5)., No change was observed in the inhibition of GJIC by TPTC alone. Thus, the inhibition of GJIC by TPTC was not mediated by PKC.

Neither GF109203X, LY294002 nor PD98059 alone at the indicated concentration had any notable effects on GJIC in these cells.

### Effects of TPTC on connexin 43 protein level and phosphorylation

**Figure 6:**
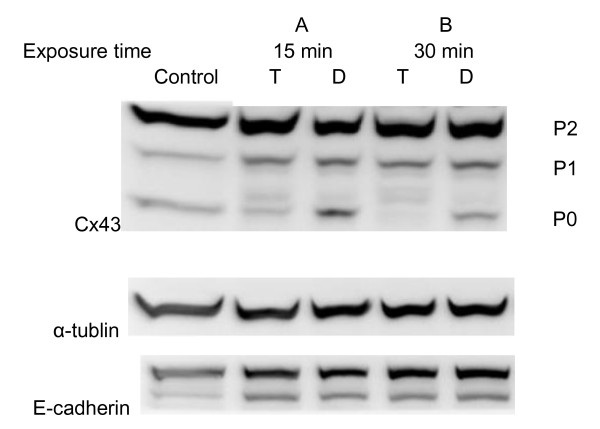
**Western blot analysis of Cx43, α-tublin, and E-cadherin protein expression alterations in TPTC treated WB F344 liver cells**. A: T, TPTC 1.5 ppm (15-min exposure); D, negative control (DMSO). B: T, TPTC 1.5 ppm (30-min exposure); D, negative control (DMSO). P0 gradually decreased and density of P1 increased. The band of P0 totally disappeared after exposure to 1.5 ppm TPTC for 30 min. No change of α-tublin, and E-cadherin protein were found. MW: Connexin 43 was 43 kD, α-Tublin was 55 kD, and E-Cadherin was 120 kD.

One possible mechanism involved in the inhibition of GJIC is abnormal phosphorylation of connexins [65-67]. WB-F433 cells express Cx43 predominantly as gap junction protein [68]. Western blot analysis was performed to detect the state of Cx43 phosphorylation in WB-F344 cells after treatment with TPTC. In untreated cells, three isoforms of Cx43, which correspond to different phosphorylated forms of Cx43, are detectable as P0 (unphosphorylated form), P1 and P2 (phosphorylated forms), respectively [69]. After 15-min and 30-min exposure to TPTC, the P0 band disappeared, and a shift to bands of higher molecular weight occurred (P1) (Fig. 6).

### Effects of TPTC on connexin 43 in immunofluorescence staining

**Figure 7:**
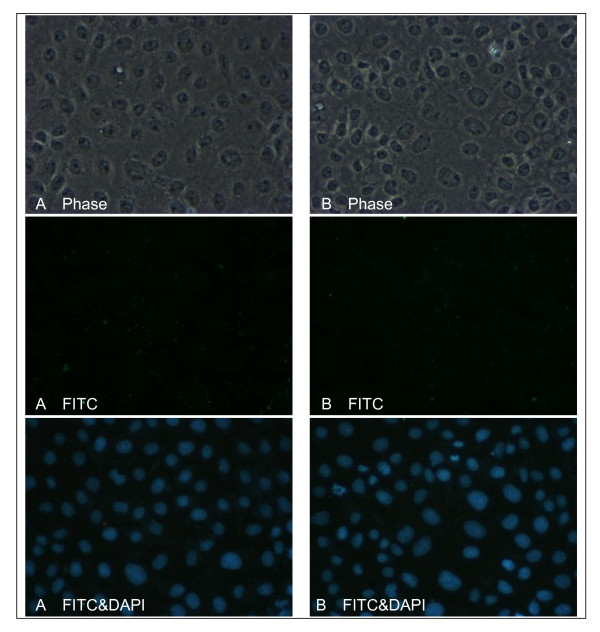
**The expression of Cx43 in WB-F344 cell under stained with FITC and DAPI**. A. expression of Cx43 in WB-F344 cell with 1.5% DMSO; B: expression of Cx43 in WB-F344 with 1.5 ppm TPTC after 30-min exposure. The fluorescent intensity did decrease in FITC stain after treatment with 1.5 ppm of TPTC for 30 min.

The expression of Cx43 in WB-F344 cell under stained with fluorescein isothiocyanate (FITC) and DAPI after 30-min exposure with1.5 ppm TPTC compared to the control group (A) with 1.5% DMSO was showed (Fig. 7). The fluorescent intensity did decrease in group (B) after exposure with TPTC.

## Discussion

Carcinogenesis is a multistep process, including "initiation," "promotion," and "metastasis" ("progression") [70]. Potter suggested that the initiation process prevents genetically altered stem cells from terminally differentiating [71], and, at the same time, GJIC restricts the growth of these cells. However, when exposed to tumor promoters, which inhibit GJIC, these transformed cells proliferate [37]. The results of this study indicate that the TPTC inhibits GJIC in WB-F344 rat liver epithelial cells in a concentration- and time-dependent manner. In the present study, we demonstrate for the first time that exposure TPTC results in downregulation of Cx43 expression in liver cell cultures. Moreover, we show that TPTC modulates Cx expression predominantly through activation of MAPK and PI3K signaling pathways. Several in vivo and in vitro studies have revealed potential effects of organotins in broad spectrum including immunosuppressive, neurotoxic, endocrinopathic, reproductive, teratogenic, developmental, and possibly carcinogenic activity [3,13,72-75]. Alterations in the phosphorylation status of connexins are a consequence of the activities of the protein kinase and/or protein phosphatases. GJIC recovered when pre-treated with PD 98059 (ERK inhibitor), and LY294002 (PI3-kinase inhibitor), but did not recover when GF109203X (Protein Kinase C inhibitor) was added. The reactions of fluorescence of Cx43 in WB-F344 cells after treatment with TPTC did decrease and the phosphorylation of Cx43 was found in Western Blot analysis. Some studies also showed that TPTC could inhibit the phosphorylation and ATP formation in chloroplasts and embryos of marine invertebrate [9,76].

The inhibition of GJIC by TPTC was independent of PKC activity but clearly dependent upon the activation of both MAPK and PI3-kinase pathways. The loss of GJIC was also described in cancer cells [77,78]. Alteration in expression of connexins may be involved in the expression of neoplastic phenotype [79] and changes in the phosphorylation pattern of connexins are also associated with GJIC inhibition by other tumor-promoting agents and oncogenes [80-82].

Hence, there is no evidence of a causal cross-talk between the two modulatory pathways, MAPK and PI3K. However, both PD58059 and LY294002 abolished completely the effect of TPTC downregulation of Cx43, implicating both MAPK and PI3K signaling cascades in a common mechanism of Cx regulation. It is possible that MAPK and PI3K act through a common downstream pathway, such as GSK-3 activation [83-86], to control endothelial cellular function through Cxs.

In conclusion, the present study shows that TPTC inhibits GJIC in WB-F344 rat liver epithelial cells by altering the Cx43 protein expression through the MAPK and PI3-kinase pathways. However, to prove the carcinogenicity of TPTC still needs further study. This preliminary study could provide the possible mechanism for further evaluation of toxicity of TPTC.

## Competing interests

The authors declare that they have no competing interests.

## Authors' contributions

CHL participated in the study design, interpretation of results, analysis, and manuscript writing. IHC participated in the study design and analysis. CRL participated in the statistical analysis and manuscript writing. CHC participated in the study design and coordination. MCT participated in the study design and coordination. JLT carried out the immunoassays, the study design, analysis and manuscript writing. HFL participated in the study design, interpretation of results and manuscript preparation. All authors read and approved the final manuscript.
